# Ironic effects of political ideology and increased risk-taking in Ohio drivers during COVID-19 shutdown

**DOI:** 10.1371/journal.pone.0279160

**Published:** 2022-12-19

**Authors:** Mason Alexander Shihab, Brittany Shoots-Reinhard

**Affiliations:** 1 Department of Psychology, The Ohio State University, Columbus, OH, United States of America; 2 School of Arts and Sciences, University of Pennsylvania, Philadelphia, PA, United States of America; 3 Center for Science Communication Research, School of Journalism and Communication, University of Oregon, Eugene, OR, United States of America; Tongji University, CHINA

## Abstract

In March 2020, Ohio, along with many other states, enacted a stay-at-home order (i.e., “shutdown”) to limit the spread of COVID-19. As a result of lower traffic, crashes should also have declined. We investigated whether crash rates declined in Ohio during the stay-at-home order and explore possible predictors for the decrease, such as reduced travel in compliance with the order, along with speeding, alcohol, and drug use. In addition, we examined whether support for President Trump would relate to greater travel and greater crashes (particularly during the stay-at-home order, when greater travel indicated lower compliance). The overall rate of crashes fell as people stayed home, mainly due to a decline in minor crashes. In contrast, the rate of serious crashes did not fall. Instead, percentage of alcohol-related crashes increased during the stay-at-home order, and the reduction in travel was associated with greater speeding-related crashes. Because alcohol and speeding tend to increase crash severity, these two factors may explain why severe crash rates were not reduced by lower traffic. Instead, it appears that those drivers remaining on the roads during the shutdown may have been more prone to risky behaviors, evidenced by a greater percentage of alcohol-related crashes across the state during the shutdown and greater speed-related crashes in counties with less traffic. In addition, county-level support for President Trump indirectly predicted greater rates of crashes (of all types) via increased travel (i.e., lower compliance with the shutdown), even while controlling for county-level income, rurality, and Appalachian region. Importantly, this mediated effect was stronger during the weeks of the shutdown, when greater travel indicated lower compliance. Thus, lower compliance with the stay-at-home order and increased risky driving behaviors by remaining drivers may explain why lower traffic did not lead to lower serious crashes.

## Introduction

### Effects of COVID-19 shutdown on traffic and crashes

To limit the spread of COVID-19, most US states enacted stay-at-home orders (i.e., “shutdowns”) in early 2020 [1] that markedly reduced traffic [2]. This gave researchers the opportunity to examine the effect of a drastic reduction in traffic on crashes. The link between rates of traffic and crashes [3, 4] suggests that reduced traffic should have resulted in reduced crashes and fatalities.

However, the expected benefit in reduction in traffic deaths did not materialize everywhere. Analyses conducted by the National Police Foundation report decreased crashes in Florida, Iowa, Ohio, Massachusetts, and Missouri in the months of March and April 2020 but also find increases in the fatality of those crashes during the same period [5]. Similarly, the North Carolina Department of Transportation shows fewer total crashes during their shutdown but no decline in fatal crashes [6], and the same was found in Connecticut [7]. The strong link between travel and crashes combined with this unfortunate durability of traffic fatalities suggests that reduced travel would indeed result in a reduction of crashes—but only those of a less serious nature.

Hypothesis 1: Reduced travel during the shutdown will be associated with lower crash rates, particularly minor crashes.

It appears that those drivers who remained on the roads post-shutdown may have engaged in riskier driving behaviors, such as speeding [8], which would increase the severity of crashes [9, 10]. Indeed, in some states (e.g., Minnesota) crashes and fatalities increased despite the reduction in traffic. Lower-than-normal traffic may have removed perceptual cues that would ordinarily reduce speeding, reduced the perceived risk of speeding, and made driving more “boring” which encourages higher speeds [11]. Additionally, stress is associated with risky driving, at least in some individuals [12], and as large numbers of Americans experienced increased anxiety and depressive symptoms, during the pandemic [13], more risky driving may also be expected.

During March and April, alcohol sales increased [14, 15] and Americans reported greater alcohol consumption [16, 17]. This is not surprising given that the use of alcohol and drugs to relieve mental health symptoms is common [18, 19], and these symptoms increased during the pandemic [13]. There is an abundance of evidence that alcohol and drugs have deleterious effects on driving, even at very low levels [20–24]. Thus, even as traffic decreased, increased substance use may have contributed to more serious crashes. However, this possibility has not yet been confirmed, so we tested the hypothesis that:

Hypothesis 2: Speeding-related, alcohol-related, and drug-related crashes would make up a larger proportion of total crashes during the shutdown than the surrounding weeks.

### Politically divided response to COVID-19

The response to COVID-19 in the United States has been starkly divided along ideological groups [25–27]. Republicans perceived less risk due to COVID-19 [25, 28, 29] and were more likely to believe that the pandemic was a hoax [30, 31]. This is unsurprising, as Trump himself called the pandemic the Democrats’ “new hoax” in late February 2020 [32]. Further studies then found that approval of President Trump drove the relationship between ideology and risk perceptions [33]. Support for and compliance with distancing behaviors to stop the spread of the virus were also strongly divided along party lines, with Democrats indicating greater support for distancing measures and complying with them at greater rates than Republicans [34]. Political differences also extended into leaving the home, and hence driving behavior: Controlling for population density, areas that voted for President Trump in 2016 engaged in less physical distancing [35] and greater mobility [36], which further indicates a reduced fear of spreading the virus. These studies suggest that responses to COVID-19 related to political party per se, rather than confounding factors such as population density (as more dense areas are both more liberal and have been more impacted by COVID-19). Thus, we expected to find that:

Hypothesis 3a: County-level support for President Trump would predict more travel, which would, in turn, predict higher crash rates.

Hypothesis 3b: The role of political ideology on crashes via travel would be stronger during the shutdown, due to lower compliance with the order among those supporting President Trump.

## Materials and methods

In the current study, we chiefly study crash rates by severity and travel frequency from cellphone data. The latter was used to estimate compliance with Ohio’s stay-at-home order. From March 23 through May 1, this order, also referred to as a “shutdown” ordered residents to stay at home unless they were engaged in an essential activity [37]. Like other states, Ohio’s traffic declined during its shutdown [38], and speeding increased during this time, in at least part of the state [39]. Because of variation over the course of a week (e.g., more traffic on weekdays), and the availability of data (e.g., cellphone mobility data were collapsed by week [40]), analyses were conducted on 22 weeks of data from March 3^rd^ to August 2^nd^. This longer timeframe was selected to establish a baseline of pre-pandemic crash rates and then to observe any lingering impacts of the shutdown. Percent of residents travelling, support for President Trump and covariates (i.e., median income, rurality, and Appalachian region, see Supplement 1 in S1 File) were available by county, so data were aggregated by county by week. Data are available at https://osf.io/2p6mh/. In addition, dashboards that summarize the main findings of this paper are available via Tableau Public at https://public.tableau.com/app/profile/mason.shihab/viz/IronicEffects/OhioCrashesReport.

### Crash data

We obtained county-level crash count data from the Ohio Department of Public Safety portal [41]. These data include descriptions of crash severity, as well as time and location. We aggregated these by week and crash severity, such that our data showed the total number of crashes as well as crashes broken down by severity level in each week. In addition, our data includes the percentage of crashes associated with speeding, alcohol, and drugs. Our analysis is concerned with crash rates in 2020, but to detect deviations from natural changes in crash rates over the course of that year, we also pulled crash data from 2015 through 2019 for comparison, as was done in a similar study in the Middle East [42], in which researchers used Z-tests to contrast the mean crash rates of crashes between 2020 and the aggregate of the prior five years.

For the data from 2015 to 2019, we then averaged each crash statistic across years, so that for each county, the weekly crash counts reflected the average numbers from those five years (for each crash type). This left us with two sets of crash rates: crashes by county by week for 2020, and the same for the average of 2015 through 2019, such that for each county and week, we had crash rates from 2020 as well as the average crash rates from the prior five years. We controlled for the five-year average crash rates in our analyses examining 2020 crash rates to better observe the relationship between crash rates and the shutdown by controlling for seasonal and other typical influences on crash rates through the year. For example, holiday weekends are associated with higher crash rates [3].

To account for population density in our analyses, crash counts were converted to their corresponding rate of crashes per 10,000 population on the county level. Because counties with lower populations would experience lower absolute numbers of crashes, we calculated the rates of crashes per ten thousand population using U.S. Census Data [43]. Some counties did not experience any crashes in a week, with the highest rate being over 9 crashes per ten thousand population (M = 3.94, SD = 1.18 in 2020).

Data also included information about the severity of crashes and whether they involved several factors, such as speeding, drug use, and alcohol use. Crashes were classified by severity into five categories in the original data, which we then reduced to three for simplicity of analysis. The crash types were property damage only (PDO), “minor injury,” “possible injury,” “serious-injury-suspected,” and “fatal.” For simplicity in our analysis, “fatal” and “serious-injury-suspected” were combined into “killed or seriously injured” (KSI). “Minor injury” and “possible injury” were combined into “injury.” Most (71.3%) of the crashes were PDO, 25.2% were Injury, and the remaining 3.4% were KSI crashes. We also analyzed number of crashes per week and percent of crashes per week due to speeding, alcohol, and drugs. For each week in our analysis, we were thus able to analyze the proportion of each crash type from all years in every county.

### Travel

To estimate movement, we utilized a publicly available cellphone mobility dataset from Cuebiq [40], a location intelligence and measurement platform. Through its Data for Good program, Cuebiq provides access to aggregated mobility data for academic research and humanitarian initiatives. This first-party data is collected from anonymized users who have opted-in to provide access to their location data anonymously, through a GDPR-compliant framework. It is then aggregated to the county level to provide insights on changes in human mobility over time. Travel was a percentage ranging from 0–1 (range = 46.7% - 83.5%, M = 69.9% (7.86%)). This statistic measures the percent of people in the county who travelled from their home address and went to another address according to Google Locations in a week.

### Shutdown

To determine the effect of stay-at-home order itself on crash rates we coded each week as either shutdown = 1, for weeks during Ohio’s stay-at-home order, or shutdown = 0 for weeks before and after the order. The stay-at-home order ran from March 23rd through April 30^th^ and falls within weeks 4 through 8 in our analysis.

### Support for President Trump

Support for President Trump was the percent of voters in a county who voted for Trump in the 2016 Presidential election according to the publicly available Ohio Secretary of State website [44]. Support was a proportion ranging from 0–1, with greater numbers indicating greater support for Trump (range = 0.31–0.81, M = 0.65, SD = 0.11).

### County rurality

County rurality was the proportion of the population living in rural areas as of the 2010 Census [43]. This is a variable that is updated with each decennial census. The rurality could range from 0 to 1, with greater numbers indicating more rural residents (range = 0.01–1.00, M = 0.48, SD = 0.25).

### Median income

Median household income was in 2018 inflation-adjusted dollars and was obtained from the U.S. Census data portal [43]. Incomes ranged from $36,894 to $104,322 (M = $53,751, SD = $10,585). To make the coefficients easier to interpret, we converted raw income to income in tens of thousands (i.e., by dividing by 10,000).

### Appalachian county

Thirty-two of Ohio’s eighty-eight counties are considered part of Appalachia by the Appalachian Regional Commission [45]. These counties were coded as a 1; non-Appalachian counties were coded as 0. This allowed region to be analogous to the other county characteristic variables, as the proportion of Appalachian county residents living in Appalachia is 1.

### Analysis strategy

Using IBM SPSS [46], we first analyze the rate of travel, and by extension, degree of compliance with the stay-at-home order while it was in effect. Next, we conducted analyses on overall crash rates and crash rates by severity. Then, to see if certain types of crashes increased, we conducted parallel sets of analyses on the percentage of weekly crashes related to speeding, alcohol, and drugs.

To test for linear and curvilinear effects of week and shutdown on the dependent variables just mentioned, we conducted generalized estimating equations (GEE) for each outcome (i.e., travel and crash rates). This approach allowed us to estimate average responses across counties, controlling for within-county correlations and analyzing all available data. In general, our approach is similar in methodology to Zhang, et al., who used a negative binomial model to identify variables that were associated with increased crash likelihood [47]. Like their analysis, our GEE approach allows for data that are not fully independent and can treat change over time as a dimension. In contrast, here, we use a linear vs. binomial model to estimate crash rates. We chose this approach because we wanted to model crash rates (as a continuous vs. categorical variable) over time controlling for county-related idiosyncrasies (e.g., differences in road conditions, traffic laws, enforcement of those laws by local law enforcement, etc.) that we could not obtain by treating county as a “subject” variable as well as other predictors (e.g., traffic, the prior 5-year crash rate) that would differ by week by county. We also chose GEE because it is robust to misspecification of the correlation model [48–50].

When crash rates were an outcome, we controlled for corresponding crash averages from the previous five years. In a separate set of analyses, we also added the “county characteristic” variables of support for President Trump, travel, rurality, income, and Appalachian region as covariates in order to quantify any explanatory impact they had on changes in crash rates over time. We selected covariates that were plausibly related to volume of traffic, compliance with the shutdown, drug and alcohol use, and crash severity. Following the recommendations of de Boer and colleagues, we retained these covariates whether or not they were significant [51], but report both models to allow for comparison with other analyses of crashes over time [5–7] and for transparency. When effects of week or shutdown are reduced by the addition of these covariates, it suggests that the observed effect of week or shutdown are in fact explained by a third variable [52], For example, we expected that amount of travel would explain lower numbers of crashes during the shutdown; an effect of shutdown that is reduced to non-significance in the presence of a significant effect of travel would be consistent with that possibility.

Because we were treating counties as N = 88 observations with 22 repeated measures (i.e., one measure per week), we estimated our power using G*Power [53] based on RMANOVA with one group and 22 measurements, a correlation of .5 among measures, .95 power, and .05 alpha. To detect a within-group effect (e.g., of week) of size f = .25, we would need a sample of 29 and for .15 effect size, we would need sample = 62. For between-factors effects (e.g., support for President Trump), we required N = 24 for .25 effect size and 60 for a .15 effect size. Thus, we concluded we would have sufficient power for our analyses.

Additionally, we conducted mediational analyses to determine whether support for President Trump indirectly predicted increased crash rates via lowered stay-home compliance. We conducted mediation analyses using PROCESS [54] within each week. The indirect effect of support for President Trump on crash rates was estimated using bootstrapping with 5,000 resamples. We controlled for the same covariates (i.e., 5-year prior average crashes, rurality, income, and Appalachian region).

## Results

### Tests of Hypothesis 1: Percent of people staying home will be associated with lower crash rates, particularly minor crashes

#### Travel

**Fig 1:**
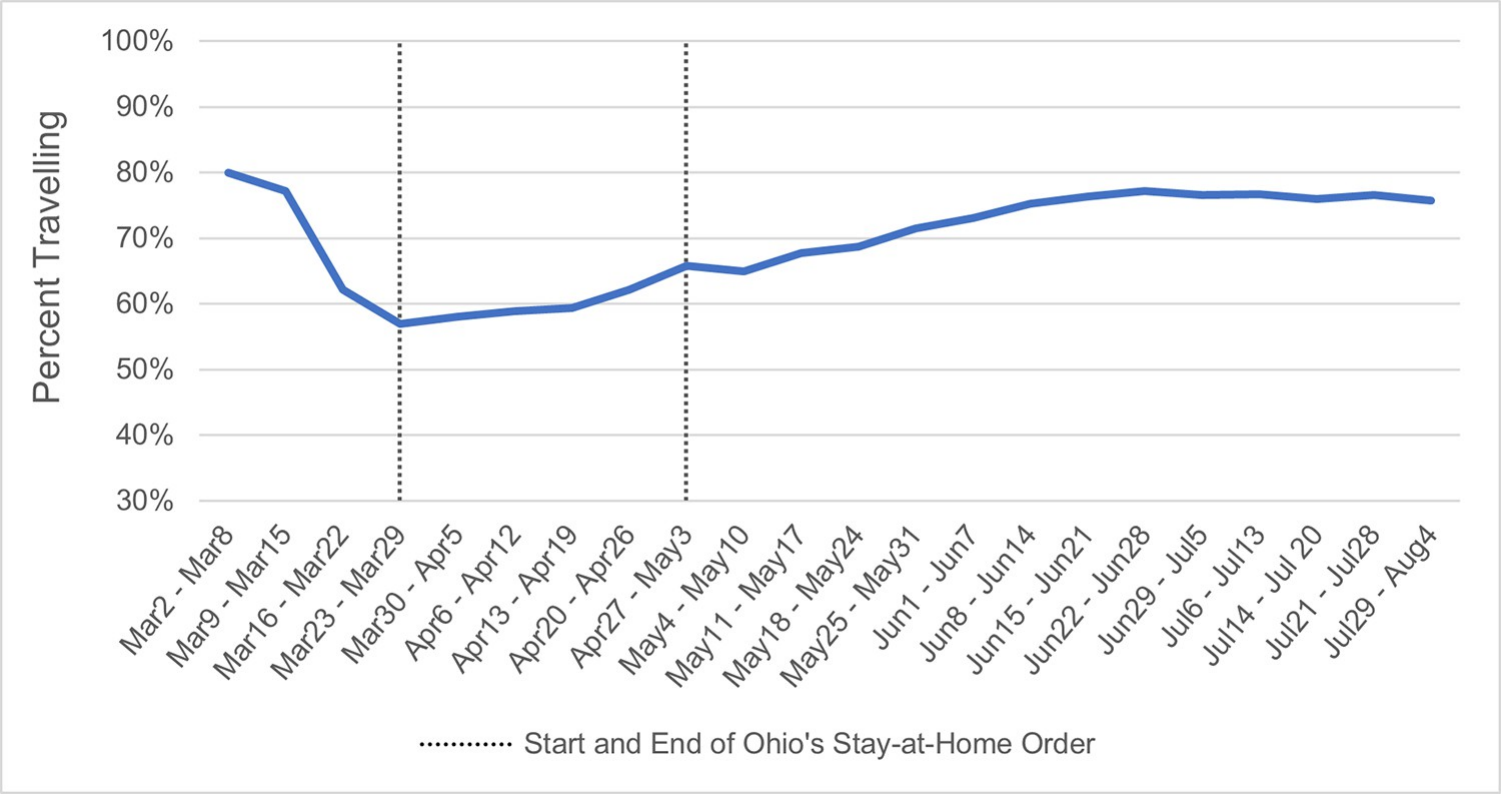
Percent of Ohioans staying home by week, adjusted for population.

Percent of people travelling was strongly affected by week and the stay-at-home order (Fig 1). Travel was significantly lower while the stay-at-home order was active, *b(se)* = −11.06 (0.19), *Wald* χ^2^(1) = 3,400.81, *p <* .001. In addition, the percentage of people travelling decreased slightly over the 22 weeks, *b(se)* = −1.04 (.04), *Wald* χ^2^(1) = 562.92, *p <* .001, and there was a curvilinear effect of week such that travelling hit a low point in late March but then increased for the remainder of the weeks we examined, *b(se)* = 0.06 (0.002), *Wald* χ^2^(1) = 908.93, *p <* .001.

In addition to the effects of week and shutdown, county characteristics also predicted rate of travel. As expected, percent of people travelling was greater for counties with more Trump voters, *b(se)* = 13.35 (1.63), *Wald* χ^2^(1) = 66.76, *p <* .001. Counties with higher median incomes had lower rates of travel, *b(se)* = −0.87 (0.14), *Wald* χ^2^(1) = 40.11 *p <* .001. Appalachian region and rurality did not predict travelling after accounting for the other predictors. The linear and curvilinear effects of week, as well as the effect of shutdown, were unchanged after adding these county characteristics covariates to the model, suggesting that the results do not depend on these additional variables.

#### Total crashes

**Fig 2:**
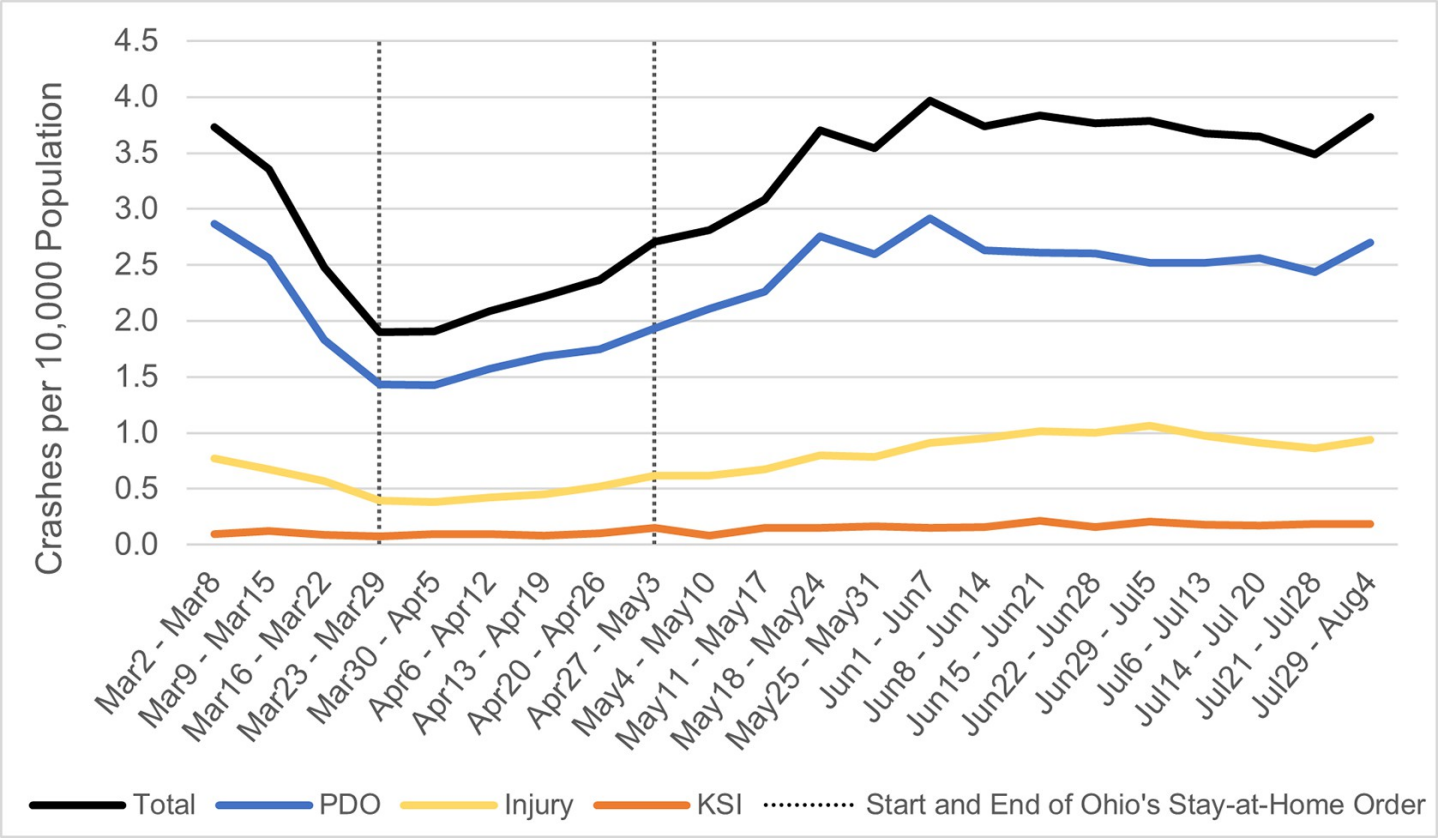
Crash rates in Ohio per week from March to August, adjusted for population.

As expected, we found that the rate of crashes was lower during the shutdown period, *b(se)* = −0.89 (0.06), *Wald* χ^2^(1) = 198.37, *p <* .001 (Fig 2). There was also a curvilinear effect of week, *b(se)* = 0.003 (0.001), *Wald* χ^2^(20.40), *p <* .001, such that crash rates fell during the first weeks of March but began to increase again throughout April. The linear effect of week was not significant, *b(se)* = −0.03 (0.01), *Wald* χ^2^ (1) = 3.48, *p =* .062. The effect of previous 5-year crash rate was significant, *b(se)* = 0.66 (0.05), *Wald* χ^2^(1) = 162.57, *p <* .001, meaning that counties that have had higher crash rates historically also had higher rates during our target period.

When we reran the analysis after the “county characteristic” variables (i.e., travel rates, proportion of Trump voters, median income, rurality, and Appalachian region) were added, the linear effect of week became significant, *b(se)* = 0.05 (0.02), *Wald* χ^2^(1) = 9.42, *p =* .002 (indicating crash rates increased over time, after accounting for additional variables). However, the effect of shutdown (*p*>.10) and the curvilinear effect of week (*p*>.05) were reduced to non-significance, as their influence on crash rates was explained by the covariates. There was an effect of traveling such that counties with more people travelling had more crashes, *b(se)* = 0.07 (0.01), *Wald* χ^2^(1) = 122.40, *p <* .001, which suggests that travel was responsible for the effects of shutdown and week on crashes. None of the other county characteristic covariates had significant effects, however, when we lastly reran this same analysis with an interaction term of rurality and shutdown, we found the interaction to be significant: *b(se)* = 0. 52 (0.24), *Wald* χ^2^(1) = 4.6, *p =* .032.

#### Crashes by severity

The effects of shutdown and week on crashes was stronger for property-damage-only (PDO) crashes than the other types. This effect is visible on Fig 2. For both PDO and injury crashes, like the total crash rate, we notice a significant curvilinear effect such that crashes first fell in late March and then resumed pre-pandemic levels by mid-June, even when controlling for the average crash rates from the past five years (**Table 1**). For KSI crashes, we did not observe a linear or curvilinear effect of week but did still find a more modest reduction during the shutdown weeks, *b(se)* = −0.03 (0.01), *Wald* χ^2^(1) = 7.42, *p =* .006.

**Table 1:** Effects of week (linear and curvilinear) and shutdown on crashes per 10,000 residents.

	PDO	Injury	KSI
Week (linear)	−0.02 (0.01)	0.00 (.001)	0.004 (0.003)
Week (curvilinear)	0.002 (.001)**	0.001 (.00) **	0.00 (0.00)
Shutdown	−0.65 (.05)***	−0.24 (.02) ***	−0.03 (0.01) **
Previous 5 year	0.62 (.047)***	−0.44 (.06) ***	0.15 (0.046) **

Parameter estimates (B) with standard errors in parentheses are reported.

*p < .05

**p < .01

***p < .001.

Importantly, the effect of shutdown was significant for all three crash types but with a noticeable decline in effect size with increasing severity. However, when county characteristic variables are added into the models, the effects of shutdown for all three crash types becomes non-significant. This result, which can be seen in **Table 2**, suggests that the effect of shutdown is being mediated by those county characteristic variables and crash rates, likely travel in particular (see Tests of Hypothesis 3). The travel rate’s effect size and significance levels appear to closely mimic the effect of Shutdown in the sparser model. With these more complete analyses, we find support for Hypothesis 1, that the proportion of people travelling would predict lower crash rates, and that this influence would be more pronounced for crashes with lower severity.

**Table 2:** Effects of week (linear and curvilinear) and shutdown, controlling for additional variables.

	PDO	Injury	KSI
Week (linear)	0.03 (.01) **	0.02 (0.01)***	0.007 (0.003)*
Week (curvilinear)	−0.001 (0.001)*	−0.001 (0.003)*	0.00 (0.00)
Shutdown	−0.10 (0.07)	−0.03 (0.03)	−0.001 (0.01)
Previous 5 year	0.57 (0.05)***	0.37 (0.06)***	0.07 (0.05)
Travel	0.05 (0.005)***	0.02 (0.002)***	0.003 (0.001)**
Median Income	−0.04 (0.03)	−0.02 (0.01)	−0.004 (0.004)
Trump Support	−0.39 (0.43)	−0.88 (0.28)**	−0.16 (0.06)*
Rural	−0.002 (0.003)	0.01 (0.01)	0.13 (0.03)***
Appalachian	−0.11 (0.09)	−0.04 (0.04)	−0.01 (0.01)

Parameter estimates (B) with standard errors in parentheses are reported.

*p < .05

**p < .01

***p < .001.

First, in addition to the effects of week on PDO crashes, counties with greater travel had higher rates of PDO crashes, *b(se)* = .05 (0.005), *p <* .001. Appalachian region, support for President Trump, rurality, and income did not have significant effects (*p*’s>.1). Analyses of minor injury crashes also supported Hypothesis 1. Independent of the effect of week, counties with more travel also had greater rates of injury crashes, *b(se)* = 0.02 (0.002), *p <* .001, as did those with lower support for President Trump, *b(se)* = −0.88 (0.28), *p =* .001. Income level, Appalachian region, and rurality did not predict minor/possible injury crash rates (*p*’s>.15). Finally, the rate of KSI crashes adjusted for population was also greater for counties with more travel, *b(se)* = 0.003 (0.001), *p =* .006, rural counties, *b(se)* = 0.13 (0.03), *p <* .001, and those with less support for President Trump, *b(se)* = -0.16(0.06), *p =* .016. Income level and Appalachian region did not predict KSI crashes (*p*’s>.50).

### Tests of Hypothesis 2: Greater proportion of speeding-, alcohol-, and drug-related crashes

As before, we first tested the linear and curvilinear effects of week, as well as shutdown, before rerunning those models with the county characteristic covariates added. We found partial support for Hypothesis 2, as there was a significant increase in alcohol-related crashes during the shutdown. More details for each crash type that we studied are below, and the full results are available at **Tables 3** and **4**.

**Table 3:** Effects of week (linear and curvilinear) and shutdown.

	Speed	Alcohol	Drug
Week (linear)	0.18 (0.17)	*−*0.002 (0.09)	*−*0.10 (0.10)
Week (curvilinear)	*−*0.004 (0.007)	0.00 (0.004)	0.002 (0.004)
Shutdown	0.90 (0.82)	1.30 (0.53)*	*−*0.30 (0.32)
Previous 5 year	0.46 (0.07)***	0.16 (0.08)*	0.06 (0.08)

Parameter estimates (B) with standard errors in parentheses are reported.

*p < .05

**p < .01

***p < .001.

**Table 4:** Effects of week (linear and curvilinear) and shutdown, controlling for additional variables.

	Speed	Alcohol	Drug
Week (linear)	−0.12 (0.19)	0.02 (0.11)	−0.08 (0.09)
Week (curvilinear)	0.01 (0.009)	−0.001 (0.005)	0.001 (0.004)
Shutdown	−2.00 (1.15)	1.59 (0.71)*	−0.10 (0.32)
Median Income	0.005 (0.39)	0.09 (0.15)	0.07 (0.13)
Trump Support	−5.50 (5.69)	2.70 (2.60)	1.26 (1.68)
Rural	6.27 (2.68)*	0.002 (0.01)	−0.71 (0.91)
Appalachian	3.80 (1.16)**	0.42 (0.50)	1.36 (0.42)**
Previous 5 year	0.31 (0.088)***	0.14 (0.07)	−0.01 (0.09)
Travel	−0.26 (0.07)***	0.03 (0.03)	0.02 (0.03)

Parameter estimates (B) with standard errors in parentheses are reported.

*p < .05

**p < .01

***p < .001.

#### Speeding-related crashes

Neither week nor shutdown predicted greater number of speed-related crashes (*p*’s>.20). Instead, counties with greater travel had a lower percentage of speed-related crashes, *b(se)* = −0.26 (0.07), *Wald* χ^2^(1) = 13.15, *p <* .001. Speed-related crashes were also higher for Appalachian counties, *b(se)* = 3.80 (1.16), *Wald* χ^2^(1) = 10.73, *p =* .001, and more rural counties *b(se)* = 6.27(2.68), *Wald* χ^2^(1) = 5.49, *p =* .019. Support for President Trump and income were not significant predictors (*p*’s>.30).

#### Alcohol-related crashes

**Fig 3:**
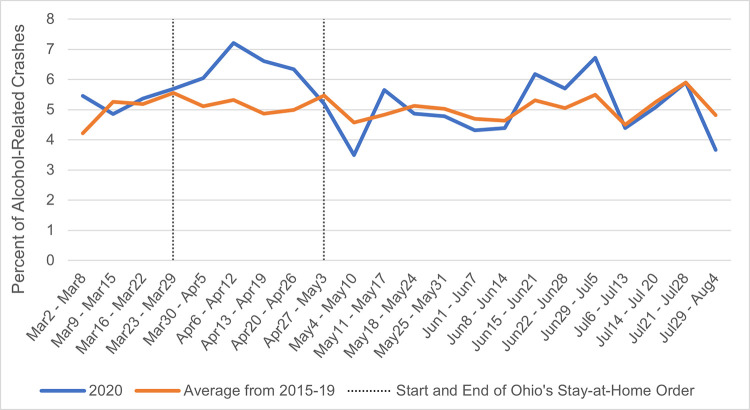
Proportion of alcohol-related crashes both from 2020 and the average of the years 2015 through 2019.

In support of Hypothesis 2, alcohol-related crashes were higher during the shutdown weeks, *b(se)* = 1.30 (0.53), *Wald* χ^2^(1) = 5.97, *p =* .015 (Fig 3). Even when the county characteristic covariates were added, the effect of shutdown remained significant *b(se)* = 1.60 (0.71), *Wald* χ^2^(1) = 5.04, *p =* .025. No other effects emerged for this crash type.

#### Drug-related crashes

The proportion of crashes related to drug use remained stable over the weeks we examined (all effects of shutdown and week p>.30). Instead, Appalachian counties had higher percentages of drug-related crashes, *b(se)* = 1.36 (0.43), *Wald* χ^2^ (1) = 10.15, *p =* .001 (and all other predictors had *p* >.40).

### Test of Hypothesis 3: Indirect effect of support for President Trump on crash rates

**Fig 4:**
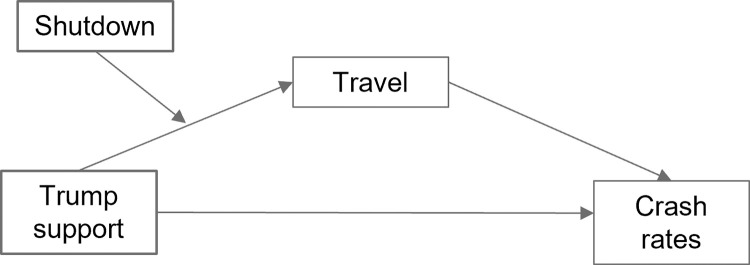
Hypothesized moderated mediation model.

Our expectation that support for President Trump would lead to greater crashes (due to lack of stay-at-home compliance) was not directly supported in our analyses on overall crash rates. However, the effect of suppressed travel could have prevented this result if the effect of ideology was fully mediated by said travel. Furthermore, we suspected the influence of Trump support on travel may be greater during shutdown weeks, as compliance with the shutdown may have depended in part on ideology. We tested this potential relationship with the moderated mediation model seen in Fig 4. To minimize likely confounds of any detected effect, we retained rurality, Appalachian county, and income as covariates.

For total crashes, the confidence interval for the index of moderated mediation of shutdown did not contain zero (*95%CI*:0.53, 1.09), indicating that the difference in conditional effects before vs. during shutdown was meaningful. In addition, the confidence interval for weeks before and after the shutdown also did not contain zero (*95%CI*:0.61, 1.24), consistent with Hypothesis 3a, and indicating that support for Trump influences greater crashes through the mediator of travel. Furthermore, this relationship strengthened during the shutdown (*95%CI*:1.45, 2.00), supporting Hypothesis 3b. This held for all the three crash types in our analysis.

The shutdown had a more modest effect on the Trump-travel-crash relation when only including specific types of crashes, but with decreasing effect sizes with more serious crashes. This aligns with decreasing effects of travel by increasing severity seen in GEE tests of Hypothesis 1. The crash type with the strongest effect was PDO (*95%CI*:0.37, 0.75). Injury was next at (*95%CI*:0.16, 0.33), and the weakest was KSI, at (*95%CI*:0.03, 0.06). As none of the indirect confidence intervals contained 0, Hypothesis 3 received strong support. See Supplement 2 in S1 File for more moderated mediation statistics and effects of covariates.

## Discussion

From the large decline in travel, we can deduce that Ohioans largely complied with the stay-at-home order, with the lowest travel in its first week (Week 4 in our analysis). Interestingly, travel had already begun to trend down before the order, perhaps due to school closings and changes to work arrangements. Ohio’s crash rates did decline briefly in April and March, closely mirroring the reduction in travel (Fig 1), but only for less serious crashes, not involving fatal or serious injuries (Fig 2). These results are consistent with our expectations based on analyses conducted on crashes during the pandemic [5–8, 47]. They are also consistent with our analyses supporting Hypothesis 1 that show a reduction in travel predicted lower crash rates (to a stronger degree with decreasing crash severity).

Travel had a greater effect on the total crash rate than it did on the rate of any single crash type (using our three-tiered approach). Thus, when travel fell during the shutdown, the overall crash rate saw a greater decline than that of PDO, Injury, or KSI crashes. This reduction was primarily driven by PDO and Injury crashes, which could be due to the relative rarity of severe crashes, even aggregated to the county level. Most counties have two or fewer severe crashes per week, so it may be difficult to detect a difference in serious crashes, given their infrequency. In addition, we did find an effect of rurality predicting more KSI crashes, perhaps due to greater distance from hospitals. For the more frequent and minor types of crashes, the reduction in travel had a stronger association with crash rate reduction (likely through reduced travel), supporting Hypothesis 1.

Hypothesis 2 was supported only for alcohol-related crashes, not for speeding or drug-related crashes. It is unclear why speed-related crashes did not increase overall during the shutdown, given the reports of greater speeding [39]. Indeed, ODOT data shows that the percent of drivers going over 85 mph on freeways increased by 50% from the start of the pandemic to July 2020, only returning to pre-pandemic levels the following April (Supplement 3 in S1 File). However, a slow increase in crashes late into the summer and delayed return to normal would not have been detected by the analyses we performed—speeding remained elevated several months after our data end. A second possibility is that there is always a link between lower amounts of traffic and higher speeds [11]. The shutdown may have indirectly contributed to speed-related crashes via reduced amount of traffic, which encourages speeding. That possibility is consistent with our findings that reduced travel, rurality, and Appalachian counties were all three independent predictors of greater speed-related crashes—all three would relate to less traffic on roads in these locations. The effects of amount of traffic and rurality are also consistent with recent findings that rural roadways are deadlier than urban roadways, due in part to speeding [55].

A related possibility is that only some areas observed increased speeding, such as those that had less travel or those that were more rural (including those located in Appalachia). Data provided by ODOT indicate that increased speeding was higher on rural freeways (Supplement 3 in S1 File) and remained high until 2021 (after our data end). It could be that in those counties in particular, people were taking advantage of empty roads with disastrous results, as some have speculated [8]. This possibility may also explain why travel has the weakest relationship with serious crashes than other crash types; while these counties may have had fewer crashes overall, the drivers remaining on the road could have been driving more dangerously and thus getting into more serious crashes.

Alcohol, but not drugs, was a factor in a greater percentage of crashes during the shutdown. While there is evidence that alcohol consumption has increased in response to COVID-19 [14, 15], the effect of COVID-19 on sales of other drugs are unknown. Although the self-medication hypothesis [18] would predict that drug consumption also increased, this may not actually be the case. The stay-at-home order may have made it more difficult to obtain illegal drugs or prompted users of legal, but mind-altering, drugs to stay home. Alcohol use is more common than illegal drug use [19], so it may have been easier to detect an effect of alcohol-related crashes increasing than drug-related crashes. Alcohol-related crashes (like speeding-related crashes) tend to be more severe [20–22], so this may explain why the reduction in traffic during the COVID-19 shutdown did not translate into lower fatalities.

Political ideology, as indicated by a county’s level of support for President Trump, was indirectly associated with more crashes via greater travel. As expected, and supporting Hypothesis 3a, the extent to which a county’s residents had voted for President Trump was related to the extent they travelled, over and above an effect of rurality, which is consistent with travel due to political noncompliance with the order rather than due to necessity. For instance, those in rural areas may be less able to stay home due to greater distances between essential businesses, but controlling for rurality and Appalachian county should have reduced the impact of this potential confounder. Furthermore, we observed this relationship controlling for median county income, which could be another indicator of travel necessity, as many lower-paid jobs (e.g., hospitality, food services, and retail) were also “essential” workers who continued to report for work in person. In addition, the indirect effect of support for President Trump on crash rates was stronger during the stay-at-home order, when travel indicated lower compliance with governmental recommendations. This last finding was consistent with our expectations and studies which found that differences in political preferences would predict differences in travel [35, 36]. It is unclear why the direct effect of Trump support predicted lower crashes when controlling for the other covariates; it suggests that some other county-level variable related to Trump support may have been unaccounted in our analyses. For example, perhaps counties with greater Trump support also have a smaller proportion of younger drivers, who are more likely to get into crashes than older drivers [56].

### Limitations

For both the rate of total crashes and all four sub-types, the effects of week remained even after controlling for travel and county characteristic variables (as well as the average rate of similar crashes over the prior five years), which suggests that additional factors contributed to the lowered crash rate. Lack of reporting may be one factor, but there may be others.

Our findings are also limited in generalizability due to our use of a convenient government data set of Ohio crashes, but future research could examine whether similar effects are observed in other states. Ohio is a good test state due to its large population and diversity in terms of our variables of interest and covariates. For example, in 2016, over 5 million Ohioans (71% of registered voters) voted in the Presidential election, and 51% of them voted for President Trump [44]. In addition, the mean rurality of Ohio counties is 48% [43].

An additional limitation of our research is the nature of the data we use. We used Ohio Department of Public Safety crash data, which is an excellent comprehensive source of information about all reported crashes in Ohio, but is limited in the extent of potential causes of crashes that can be identified. Speed and substance use are both objective contributors to crashes included in reports, but crash reports are less useful for causes that are more difficult to prove and therefore underreported, such as driver distraction [57]. In addition, the analysis we conducted, which due to availability of many variables, was at the level of county rather than at the level of the individual driver. The gold standard for studying the effects of driver behavior on crashes would be to use telematics or camera systems to monitor drivers and identify behaviors directly preceding crashes, such as the Naturalistic Driving Study conducted by Virginia Tech Transportation Institute [58, 59]. Future research using these data or similar data could examine whether individual participants who drove during the pandemic engaged in riskier behaviors than before and estimate the effect of traffic on individual driver behavior, which appeared to be a key contributor to speed-related crashes.

### Practical applications

Our analysis presented a strong case for the existence of a political difference in response to significant public safety measures, which has been noted elsewhere [25–27, 34–36]. However, our results reveal several novel ironic consequences for the shutdown order on lower traffic accidents. While the shutdown kept people off the road, it was also associated with greater alcohol-related crashes and speed-related crashes, which tend to be more serious (Supplement 3 in S1 File) [9–10, 20]. Thus, most of the benefit for reduced traffic on crashes were for less-serious ones. This benefit was only temporary, as it was likely due to time-limited travel restrictions. However, the ironic consequences may outlast and outweigh these benefits, as increase rates of serious crashes and speeding violations persisted, even as traffic increased to normal [60].

Researchers should continue to examine the relationship between political ideology and decisions in response to government policy and the consumption of information. Although we focused on traffic here, there may be other indirect consequences, such as increased rates of alcohol-related diseases [61]. This, combined with our results, should also direct researchers to investigate the effect of these elevated figures beyond traffic accidents.

## Conclusion

In this paper we analyzed publicly available data to assess the impact of government orders to stay home on actual travel and the resulting effect on crash rates, as well as the influence of other factors, such as political ideology. Most notably, we find a much stronger reduction in less serious crashes than more serious ones as well as increases in speeding and alcohol use among those who remained (and crashed) on the road. These unintended effects could be considered “ironic,” as they manifest as results of public health orders but are themselves deleterious to public health. In addition to increased speeding and alcohol use, we posited Trump support as a possible reason for additional crashes via reduced compliance with the stay-at-home order and found an indirect effect of Trump support on increased crashes via traffic that was further moderated by the presence of the shutdown. Taken together, the results suggest that lower compliance with the stay-at-home order and increased risky driving behaviors by non-compliant drivers may explain why lower traffic did not lead to lower serious crashes.

## Supporting information

S1 File Supporting information in single file. Includes information about covariates, moderated mediation tables, Ohio speeding by rural vs. urban location, correlation table.
(DOCX)
