# Supplementary material for: Ironic effects of political ideology and increased risk-taking in Ohio drivers during COVID-19 shutdown
Source: PLoS One. 2022 Dec 19;17(12):e0279160. doi: 10.1371/journal.pone.0279160 (PMC9762566; doi:10.1371/journal.pone.0279160)
Supplement: S1 File — Includes information about covariates, moderated mediation tables, Ohio speeding by rural vs. urban location, correlation table. (DOCX) [file pone.0279160.s001.docx]

**Supporting information for**

**Ironic effects of political ideology and increased risk-taking in Ohio drivers during COVID-19 shutdown**

**Mason Shihab and Brittany Shoots-Reinhard**

Contents

[**Supporting information for** 1](#_Toc121316655)

[**Supplement 1: Covariates** 2](#_Toc121316656)

[**Supplement 2. Moderated Mediation Statistics** 3](#_Toc121316657)

[**Supplement 3. Ohio Department of Transportation Safety Briefing on Speeding [49]** 4](#_Toc121316658)

[**Supplement 4: Correlations between key variables.** 5](#_Toc121316659)

**Supplement 1: Covariates**

In addition to support for President Trump and percent of Ohioans travelling, we examined whether the proportions of crashes might be related to three other variables related to the impact of COVID-19. The first is rurality. Urban areas support President Trump less, have more cases than rural areas, and more ability to stay closer to home than people in rural areas, so we expected rural areas to stay home less. In addition, crashes in rural counties are further from hospitals on average than crashes in urban centers, which could make fatalities more likely.

Second, we examined income. Income may mitigate the impact of COVID-19 on economic and health outcomes, so we expected that income might reduce the proportion of drug- and alcohol-related crashes. Finally, we included Appalachian region. Thirty-two of Ohio’s eighty-eight counties are considered part of Appalachia by the Appalachian Regional Commission [41].

Counties in Appalachian Ohio tend to have lower income, higher poverty, and lower educational attainment [37]. Appalachian Ohio has worse health outcomes than non-Appalachian Ohio, including higher rates of drug use, but less excessive drinking [40]. Because of the possibility of greater economic and health impacts on Appalachia in particular, we thought that alcohol and drug-related crashes may be disproportionately high for that region. Including these variables was also important to help us distinguish between the effects of support for President Trump versus potential confounds, such as county level of objective COVID-19 risk and impact.

**Supplement 2. Moderated Mediation Statistics**

**Table S1. Results of Moderated Mediation Analyses. Direct, Indirect, and Moderated Effect Sizes with standard errors in parentheses are reported. *0∉[95%CI]**

|  | KSI | Injury | PDO | *Total Crashes* |
| --- | --- | --- | --- | --- |
| Direct Effect of Trump Support | −0.17(0.06)* | −0.91(0.14)* | −0.21(0.27) | *−1.09(0.33)** |
| Indirect Effect of Trump Support via Travel, Shutdown=0 | 0.04(0.01)* | 0.28(0.05)* | 0.61(0.11)* | *0.91(0.16)** |
| Indirect Effect of Trump Support via Travel, Shutdown=1 | 0.09(0.01)* | 0.52(0.05)* | 1.17(0.10)* | *1.72(0.14)** |
| **Index of Moderated Mediation** | **0.04(.01)*** | **0.24(0.04)*** | **0.56(0.10)*** | ***0.81(0.14)**** |

**Table S2. Results of Moderated Mediation Analyses. Direct, Indirect, and Moderated 95% Confidence Interval lower and upper bounds are reported. *0∉[95%CI]**

|  | KSI | Injury | PDO | *Total Crashes* |
| --- | --- | --- | --- | --- |
| Direct Effect of Trump Support | [−0.30, −0.05]* | [−1.19, −0.63]* | [−0.75, 0.32] | *[−1.74, −0.44]** |
| Indirect Effect of Trump Support via Travel, Shutdown=0 | [0.03, 0.06]* | [0.19, 0.38]* | [0.40, 0.84]* | *[0.61, 1.24]** |
| Indirect Effect of Trump Support via Travel, Shutdown=1 | [0.06, 0.11]* | [0.43, 0.61]* | [0.99, 1.37]* | *[1.45, 2.00]** |
| **Index of Moderated Mediation** | **[0.03, 0.06]*** | **[0.16, 0.33]*** | **[0.37, 0.75]*** | ***[0.53, 1.09]**** |

**Supplement 3. Ohio Department of Transportation Safety Briefing on Speeding [49]**


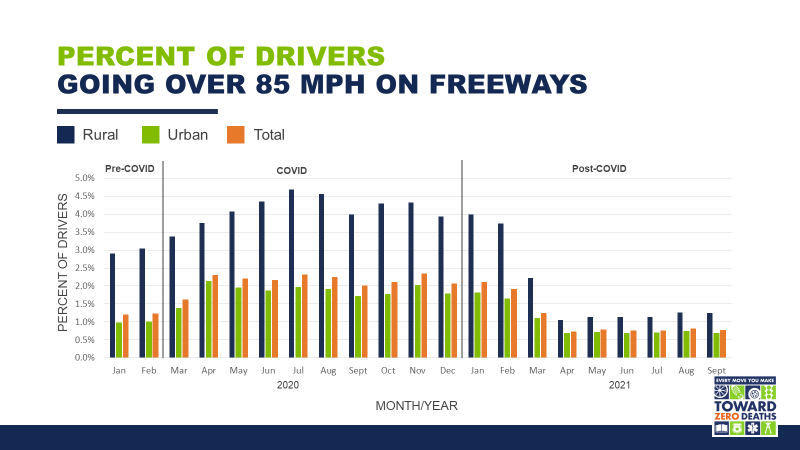


**Supplement 4: Correlations between key variables.** All crash variables are 2020 rates. Alcohol-related, drug-related, and speeding-related crashes are a percentage of total crashes per week per county. PDO=Property Damage Only; KSI=Killed or Seriously injured. Total crashes, PDO, Injury, and KSI crashes are adjusted by county population (per 10,000 residents).

|  |  | 1 | 2 | 3 | 4 | 5 | 6 | 7 | 8 | 9 | 10 | 11 |
| --- | --- | --- | --- | --- | --- | --- | --- | --- | --- | --- | --- | --- |
| 1 | Trump Support |  |  |  |  |  |  |  |  |  |  |  |
| 2 | Median income | −.07^**^ |  |  |  |  |  |  |  |  |  |  |
| 3 | Appalachian County | .20^**^ | −.52^**^ |  |  |  |  |  |  |  |  |  |
| 4 | Rural County | .74^**^ | −.28^**^ | .43^**^ |  |  |  |  |  |  |  |  |
| 5 | Travel | .21^**^ | −.13^**^ | .10^**^ | .20^**^ |  |  |  |  |  |  |  |
| 6 | Alcohol-Related Crashes | .06^**^ | −.02 | .05^*^ | .06^**^ | −.04 |  |  |  |  |  |  |
| 7 | Drug-Related Crashes | .03 | −.04 | .10^**^ | .04 | .01 | .24^**^ |  |  |  |  |  |
| 8 | Speed-Related Crashes | .07^**^ | −.13^**^ | .24^**^ | .17^**^ | −.02 | .12^**^ | .17^**^ |  |  |  |  |
| 9 | Total Crashes | −.13^**^ | −.10^**^ | −.11^**^ | −.14^**^ | .48^**^ | −.04 | −.05^*^ | .02 |  |  |  |
| 10 | PDO Crashes | −.10^**^ | −.08^**^ | −.14^**^ | −.16^**^ | .41^**^ | −.08^**^ | −.09^**^ | −.03 | .93^**^ |  |  |
| 11 | Injury Crashes | −.16^**^ | −.08^**^ | −.01 | −.09^**^ | .37^**^ | .02 | .01 | .07^**^ | .69^**^ | .41^**^ |  |
| 12 | KSI Crashes | .06^**^ | −.07^**^ | .06^*^ | .13^**^ | .18^**^ | .12^**^ | .10^**^ | .12^**^ | .26^**^ | .08^**^ | .15^**^ |

*p<.05, **p<.01, ***p<.001
